# The Puzzles Test and the Red Shapes Test as new diagnostic tools for neglect syndrome

**DOI:** 10.1192/j.eurpsy.2022.2281

**Published:** 2022-09-01

**Authors:** V. Propustina, G. Stepanov, D. Yurina, N. Varako, M. Kovyazina, S. Vasilyeva, V. Daminov

**Affiliations:** 1 Lomonosov Moscow State University, Faculty Of Psychology, Moscow, Russian Federation; 2 Research Center of Neurology, Department Of Neurorehabilitation And Physiotherapy, Moscow, Russian Federation; 3 Pirogov National Medical and Surgical Center, Medical Rehabilitation Clinic, Moscow, Russian Federation

**Keywords:** neuropsychological assessment, spatial neglect, unilateral neglect, visuospatial search

## Abstract

**Introduction:**

Neuropsychological methods for diagnosing neglect syndrome (NS) are focused on identifying the inability of patients to respond to stimuli localized in contralesional space. There are a large number of methods capable of diagnosing spatial neglect, but at the same time having various limitations and restrictions in their use.

**Objectives:**

To devise and to test universal diagnostic techniques for visuospatial neglect detection.

**Methods:**

1) A.R. Luria test battery; Trail Making Test (Part A); the Bells Test; 2) Authors’ methods: the Puzzles Test, the Red Shapes Test. A total of 47 patients after stroke with right hemisphere damage participated in the study and were divided into a target (18 patients with NS) and a control (29 patients without NS) groups. The Puzzles Test consists of three tasks: turning over cards, completing a sentence using cards with letters, completing a picture. The Red Shapes Test consisted in the search for a variable number of geometric shapes. Objective indicators of the study: total task completion time, the number of left omissions.

**Results:**

The sensitivity of the tests to NS was examined using the Mann-Whitney U-test. Differences in the number of omissions and task completion time between patients with and without spatial neglect were statistically significant regarding all tasks: turning over cards (p=0.01), completing a sentence (p<0.001), completing a picture (p<0.001), finding geometric shapes (p<0.01).

**Conclusions:**

The Puzzles Test and Red Shapes Test along with the foreign tests (the Bells Test, Trail Making Test) are sufficiently effective methods for spatial neglect detection.

**Disclosure:**

No significant relationships.

